# The Relationship Between Body Posture and Psychophysical Functioning in Children with Obesity: A Narrative Literature Review and Future Research Perspective Related to Preliminary Research Concept

**DOI:** 10.3390/medicina62040779

**Published:** 2026-04-17

**Authors:** Kornelia Korzan, Kamila Czepczor-Bernat, Paweł Matusik, Anna Brzęk

**Affiliations:** 1 Department of Physiotherapy, Faculty of Health Sciences in Katowice, Medical University of Silesia, 40-055 Katowice, Poland; abrzek@sum.edu.pl; 2 Doctoral School, Medical University of Silesia, 40-055 Katowice, Poland; 3 Department of Pediatrics, Pediatric Obesity and Metabolic Bone Diseases, Faculty of Medical Sciences in Katowice, Medical University of Silesia, 40-055 Katowice, Poland; kamila.czepczor-bernat@sum.edu.pl (K.C.-B.); pmatusik@sum.edu.pl (P.M.)

**Keywords:** childhood obesity, body posture, psychophysical functioning

## Abstract

Childhood obesity is a growing global health problem with significant biomechanical and psychosocial consequences. While many studies have examined these domains separately, few integrate postural abnormalities, psychophysical functioning, and lifestyle factors within a single framework. This narrative review synthesises the literature published between 2005 and 2025 to summarise current evidence and identify research gaps. The findings indicate that overweight and obesity increase the risk of musculoskeletal deviations such as genu valgum, flat feet, and increased lumbar lordosis, as well as altered gait biomechanics and reduced motor competence. Excess body weight is also associated with lower self-esteem, negative body image, depressive symptoms, and reduced health-related quality of life in children and adolescents. These outcomes appear to be influenced by modifiable lifestyle factors, including parental health behaviours, sleep patterns, and screen time, although reported associations remain inconsistent. Notably, few studies address biomechanical, psychological, and environmental factors simultaneously, which limits the understanding of their interactions. To address this gap, a prospective observational study of 250–300 children aged 7–17 years is proposed. The study will combine objective postural assessments, validated psychometric tools, and lifestyle analyses at baseline and after a 12–14-month follow-up. This integrated approach aims to identify postural compensation patterns, psychosocial risk trajectories, and modifiable behavioural predictors associated with childhood obesity, supporting the development of early preventive and interdisciplinary interventions.

## 1. Introduction

Childhood obesity has emerged as one of the most pressing public health challenges, affecting both Poland and the global population [1,2]. Its prevalence has been steadily increasing, raising significant concern given its long-term health and social implications. It is estimated that up to one in four school-aged children has excess body weight. Data from the most recent edition of the Childhood Obesity Surveillance Initiative (COSI, 2022–2024) indicate that among children aged 7–9 years, approximately 25% are overweight, while 11% are classified as obese [3]. Moreover, the prevalence of overweight varies substantially between countries, ranging from 9% to as high as 42%, while obesity prevalence ranges from 3% to 20%, underscoring the complexity and multifactorial nature of the problem. Since 2007, the Childhood Obesity Surveillance Initiative (COSI), conducted under the auspices of the World Health Organization (WHO), has generated essential data on trends in overweight and obesity among children aged 7–9 years. In addition to anthropometric measurements, the initiative assesses dietary habits, physical activity levels, sedentary behaviours, and both familial and school environmental factors, thereby facilitating a comprehensive understanding of obesity-related risk factors and their potential long-term consequences. Childhood obesity is a complex, multifactorial disease in which the core mechanism is an excess of energy intake relative to energy expenditure, interacting with a wide range of biological, genetic, metabolic, psychosocial, and environmental factors. The increasing prevalence of childhood obesity is further corroborated by a substantial body of research demonstrating its association not only with serious metabolic disturbances but also with clinically significant changes in the musculoskeletal system [4]. In children with obesity, a higher prevalence of postural deviations, weakened core stabilisation, diminished motor competence, and an increased vulnerability to musculoskeletal pain has been reported [5,6,7]. Existing research highlights the importance of incorporating developmental and allometric factors when interpreting physical performance in children, as growth and maturation significantly influence motor outcomes and their assessment [8]. Concurrently, a growing body of research has focused on the psychological and psychosocial dimensions of functioning among children and adolescents with excess body weight, such as lowered self-esteem, emotional problems, and body image dissatisfaction [9,10]. Against this backdrop, elucidating the relationships between obesity, body posture, and psychophysical functioning in children and adolescents is of particular importance. Although the existing literature provides numerous valuable insights, significant research gaps persist, particularly in terms of integrative and interdisciplinary perspectives. This review aims to synthesize current evidence, outline the principal mechanisms underlying these associations, and identify directions for future research.

## 2. Materials and Methods

A literature search of articles published in English was conducted via the Web of Science and PubMed databases between August and December 2025. The narrative review covered publications from 2005 to 2025. Inclusion criteria comprised studies involving children with overweight or obesity, with particular emphasis on disturbances in body posture and assessments of psychophysical functioning. Only studies with full-text availability (e.g., observational studies, quantitative studies, systematic reviews, and meta-analyses) were considered. Additionally, publications such as editorials, letters, author responses, and review articles were excluded, as were studies without accessible full texts. The initial search identified 380 records, from which duplicate entries were removed. Following abstract screening, 20 articles were deemed eligible for full-text evaluation. This review was not registered in any formal registry. The search strategy employed the following keywords: “body posture,” “childhood obesity,” and “psychophysical health.” The age range of the children was not specified. The narrative literature analysis revealed a lack of explicit integration of these domains; however, three recurrent research areas were identified across the reviewed publications. The first encompasses the biomechanical consequences of obesity, including alterations in the musculoskeletal system, postural abnormalities, and limitations in motor performance. The second area addresses the psychological and social consequences of excess body weight, such as reduced self-esteem, emotional difficulties, and impaired quality of life. The third focuses on the role of the family environment and the formation of lifestyle habits, including physical activity, dietary behaviours, and caregivers’ health awareness. Based on the findings of the review, the framework of an original prospective observational research project was developed, integrating the assessment of somatic, biomechanical, and psychosocial parameters in children and adolescents with excess body weight. This multidimensional model enables the exploration of complex relationships between obesity, body posture, and psychophysical functioning, thereby constituting a meaningful contribution to the existing body of literature.

## 3. Results

### 3.1. Results of the Narrative Literature Review

In children and adolescents, excess body weight leads to substantial biomechanical consequences, including increased mechanical loading of the locomotor system, altered gait biomechanics, and excessive stress on soft tissue structures such as articular cartilage, tendons, and fascia, which may contribute to the development of degenerative processes and musculoskeletal dysfunction [11]. Overweight and obesity are important contributing factors to the development of abnormalities in body posture [12]. A markedly higher prevalence of postural abnormalities has been reported among children with obesity compared with children with normal body weight [13].

A meta-analysis by Pablo Molina-García et al. demonstrated that children with overweight and obesity have a markedly increased risk of genu valgum compared with normal-weight peers (RR = 5.92), as well as elevated risks of flatfoot (RR = 1.49) and increased lumbar lordosis (RR = 1.41) [12]. An analysis of 73 studies provides robust evidence linking excess body weight in childhood to postural abnormalities and altered gait biomechanics, highlighting the importance of early screening and preventive strategies in paediatric populations.

Katarzyna Maciałczyk-Paprocka et al. assessed the prevalence of postural abnormalities among children and adolescents with overweight and obesity in a local population-based study conducted in Poland. The study included a representative sample of 2732 children aged 3–18 years, corresponding to 3.5% of the city’s paediatric population within this age range. Among children with excess body weight, a significantly higher prevalence of postural defects was observed, amounting to 69.2% in children with overweight and 78.6% in those with obesity, compared with peers with normal BMI. The most frequent and statistically significant abnormalities—consistent with findings from the aforementioned meta-analysis—affected the lower limbs, particularly genu valgus and flatfoot. These results indicate that overweight and obesity substantially increase the risk of developing postural disorders in children, underscoring the need for early identification and the implementation of targeted preventive strategies [5]. In children with obesity, excessive loading of the locomotor system and altered weight transfer to the lower limbs affect gait biomechanics, leading to numerous adaptive and compensatory changes. A study by Larios-Tinoco et al. demonstrated a statistically significant correlation between increased body mass and greater step width (r = 0.41, *p* < 0.001), which serves as a compensatory mechanism to maintain stability during locomotion. Furthermore, reductions in step length and swing phase velocity are commonly observed, indicating impaired locomotor capacity and a tendency to optimize energy expenditure during gait [14].

Beyond biomechanical parameters, the psychosocial dimension constitutes an integral component of health assessment in children and adolescents with obesity. Obesity during the developmental period is associated not only with increased loading of the musculoskeletal system but also with reduced self-esteem, body image dissatisfaction, and an elevated risk of mood disorders, including depression and anxiety. Incorporating this dimension enables a more comprehensive understanding of child functioning, encompassing physical health, psychological well-being, and social relationships.

The relationship between obesity and the risk of depressive and anxiety disorders is multifactorial and may vary by sex, as well as individual psychological and environmental factors. Findings from prospective studies, including analyses by Anderson et al., indicate that obesity during adolescence may significantly increase the risk of developing depressive and anxiety disorders in girls. In a study with over 20 years of follow-up, obesity between the ages of 9 and 18 years was associated with an almost fourfold increased risk of major depressive disorder (MDD) and a comparable increase in the risk of anxiety disorders in girls. In contrast, no statistically significant associations between obesity and subsequent development of depression or anxiety disorders were observed in boys [9].

Recent studies, including systematic reviews and meta-analyses, indicate consistent associations between obesity and the risk of depressive disorders. A meta-analysis by Rao et al., involving 69,893 participants, demonstrated that children and adolescents with obesity are significantly more likely to experience depressive episodes compared with their normal-weight peers (OR = 1.85; 95% CI: 1.41–2.43). These findings suggest that excess body weight constitutes an important risk factor for the development of clinically significant depressive symptoms in paediatric and adolescent populations [15].

Comparable conclusions were reported in a meta-analysis published in 2024 by Chen et al., which included 22 observational studies comprising a total of 175,135 participants. The analysis confirmed a significant association between obesity and the risk of developing depression (RR = 1.32; 95% CI: 1.09–1.60). Additionally, an elevated risk of depressive symptoms was observed among children and adolescents with obesity (RR = 1.16; 95% CI: 1.00–1.35). Subgroup analyses indicated that this association was particularly pronounced in populations from Western countries, potentially reflecting distinct cultural determinants, greater societal pressure related to body image, and specific environmental factors [16].

Another important psychosocial dimension in children and adolescents with excess body weight is reduced self-esteem and dissatisfaction with body image. In a meta-analysis of 28 studies, Moradi et al. demonstrated that obesity was significantly associated with an increased risk of low self-esteem (pooled RR = 1.53; 95% CI: 1.16–2.02), whereas overweight was linked to a smaller, yet still significant, increase in risk (RR = 1.15; 95% CI: 1.00–1.31). Furthermore, obesity was strongly associated with body image dissatisfaction (RR = 4.05; 95% CI: 2.34–7.02), while no significant association was observed for overweight [10].

In the study Obesity in Childhood and Adolescence: The Role of Motivation for Physical Activity, Self-Esteem, Implicit and Explicit Attitudes toward Obesity and Physical Activity, Scotto di Luzio et al. reported that participants with normal body weight exhibited significantly higher levels of global self-esteem and superior sport-related competencies compared with those with overweight or obesity, despite participation in a structured physical activity program lasting approximately 8–10 months [17].

A Dutch study conducted by the Centre for Overweight Adolescent and Children’s Healthcare (2023) demonstrated that health-related quality of life (HRQoL) in children and adolescents with excess body weight declines significantly with increasing obesity severity, with the most pronounced impairments observed among individuals with severe (morbid) obesity. In this subgroup, markedly poorer outcomes were reported across both physical and psychological domains, reflecting a substantial burden of symptoms that limit daily functioning and psychoemotional health. Furthermore, reduced scores were observed in the domains of autonomy and parent–child relationships, suggesting greater difficulties related to family support and independent coping abilities. Notably, low concordance was found between children’s self-reported HRQoL and parental proxy assessments, with caregivers consistently rating their children’s quality of life as lower, particularly in psychological domains. These discrepancies highlight the importance of integrating both patient-reported and caregiver-reported outcomes in clinical evaluation and in the design of therapeutic interventions aimed at improving quality of life in paediatric obesity [18].

The family environment constitutes a fundamental factor shaping a child’s health-related behaviours. Parental attitudes toward nutrition, sleep, and the use of electronic devices directly translate into children’s everyday practices. As primary behavioural role models, parents influence lifestyle patterns through meal organization, regulation of daily routines, and control of screen time. A lack of structure in these domains may promote the development of overweight, whereas consistent health-promoting practices strengthen self-regulatory capacities and support obesity prevention during the developmental period. Structural equation modelling (SEM) analyses conducted by Garcia-Conde et al. identified two key behavioural pathways. Longer sleep duration, resulting from consistent family rules and positive attitudes toward sleep hygiene, was associated with lower child BMI. In contrast, greater availability of electronic devices and a more permissive approach to screen use led to increased television viewing time, which in turn was associated with a higher BMI. Although parental attitudes influenced the home food environment and a child’s healthy habits, dietary behaviours did not demonstrate a direct association with BMI [19]. A meta-analysis by Ju Suk Lee et al. demonstrated a significant global association between parental weight status and the risk of overweight or obesity in children. Offspring of parents with overweight or obesity exhibited nearly a twofold higher risk of these conditions (OR = 1.97; +97%), underscoring the critical role of both genetic predisposition and the shared family environment, including dietary habits and levels of physical activity, in shaping obesity risk in paediatric populations [20]. Supplementary Table S1 presents a summary of the articles included in the analysis.

### 3.2. Future Research Perspective—Own Preliminary Research Concept

Based on the findings obtained from the analysis of the previously described studies, a research project entitled Body Posture in Children and Adolescents with Obesity from a Psychophysical Perspective was developed, adopting an interdisciplinary approach to the diagnosis and management of obesity.

The project aims to provide a multidimensional assessment of functioning in children with excess body weight, encompassing biomechanical, psychological, and environmental domains. The planned study population will include approximately 250–300 children and adolescents aged 7–17 years, comprising an experimental group with obesity and an age- and sex-matched control group with normal body weight. The study is designed as a prospective, observational investigation with assessments conducted at two time points: at baseline and after 12–14 months, enabling the evaluation of changes in participants’ physical and psychological functioning over time.

Body posture will be assessed using instruments such as a scoliometer, an inclinometer, and the Matthias and Thomayer tests. Psychological functioning will be evaluated using validated questionnaires, including SENA (the validated Polish version of a comprehensive tool assessing emotional and behavioural functioning in children and adolescents across developmental stages), KIDSCREEN-27, and PAQ-C/A. The primary outcome will be changes in postural alignment parameters, including spinal asymmetry, trunk rotation angle, thoracic kyphosis, and lumbar lordosis, measured using a Bunnell scoliometer, a Saunders TMX-127 inclinometer, and a Rippstein plurimeter. Secondary outcomes will include changes in psychophysical functioning, as assessed by SENA and KIDSCREEN-27 scores, as well as BMI z-score. Additionally, the analysis of children’s lifestyle behaviours and caregivers’ health awareness will enable the identification of associations between parental behaviours and children’s postural alignment and psychophysical functioning. Follow-up assessment will allow for the evaluation of changes over time, including the impact of lifestyle factors and parental health awareness on disease progression, as well as the identification of potential compensatory patterns within the musculoskeletal system.

Statistical analysis will be conducted using mixed-effects models, with a 2 × 2 design: group (control vs. obesity) × time (baseline vs. follow-up).

The primary objectives of the project include determining the prevalence and characteristics of postural disorders in children with obesity, as well as analysing the relationships between postural abnormalities and psychophysical functioning, including self-esteem, body image, and quality of life, based on correlation analyses using baseline measurements. Additionally, the project aims to examine the influence of lifestyle factors and parental health awareness on the development of postural disorders and children’s psychological functioning using multiple linear regression based on baseline and 12-month follow-up data.

**Figure 1:**
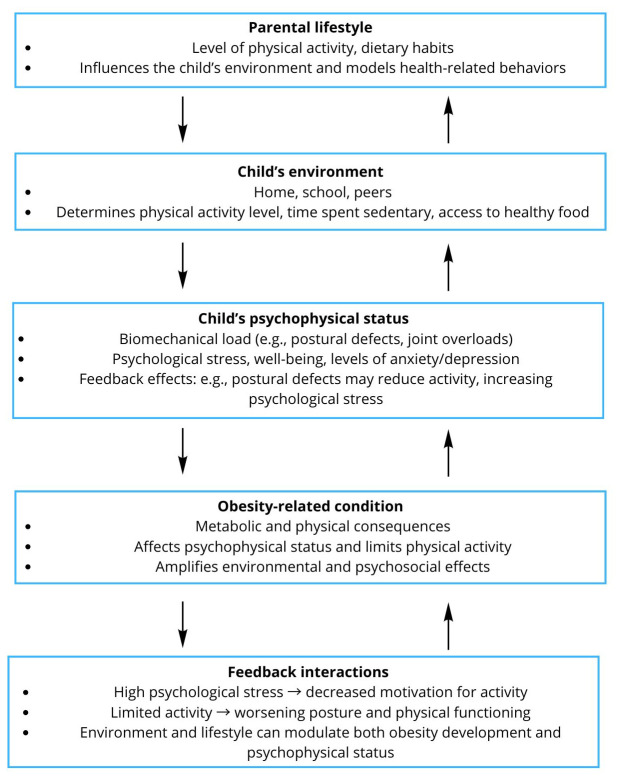
Conceptual model illustrating the relationships between obesity, body posture, psychophysical functioning, and environmental factors in children, showing their interactions.

The project is innovative in nature, as it integrates objective measurements of body posture with assessments of mental health, quality of life, and lifestyle factors in children and adolescents with obesity. The use of standardized clinical instruments, functional tests, and validated psychometric questionnaires will enable a more comprehensive evaluation of the health status of the study population. The obtained results may contribute to the development of preliminary preventive and therapeutic recommendations, support the identification of children requiring early intervention, and thereby enhance the prevention of obesity-related complications and improve quality of life in young patients while also addressing the existing research gap and providing novel scientific insights. A schematic diagram illustrating the relationships described in this section is presented in Figure 1 (own elaboration).

## 4. Discussion

Current epidemiological research confirms that the prevalence of overweight and obesity among children and adolescents is not stabilising but continues to increase globally. Systematic reviews and meta-analyses encompassing data from the past two decades indicate that this trend affects both high- and middle-income countries, with the highest prevalence observed in nations with advanced economic development. Key risk factors include insufficient physical activity, excessive screen time, and other environmental and behavioural determinants. Moreover, childhood obesity is associated with an increased risk of comorbid conditions affecting both physical and mental health, including arterial hypertension and mood disorders [21].

These data suggest that the number of children and adolescents affected by the consequences of obesity—including postural disorders, biomechanical and metabolic complications, and psychosocial disturbances—is likely to continue to rise. This trend underscores the urgent need for coordinated public health action, as well as for research aimed at identifying underlying risk mechanisms and delineating populations that require early preventive and therapeutic interventions.

Numerous preventive programmes and movement-based interventions targeting children with overweight or obesity, grounded in various forms of physical activity, have been shown to confer benefits for body posture, fundamental movement patterns, and overall physical fitness. Research indicates that such interventions exert a significant positive effect on postural alignment, gait mechanics, and physical capacity in children with excess body weight [22,23]. In a cohort of children and adolescents with excess body weight, Scotto di Luzio et al. observed a significant negative correlation between explicit (declarative) and implicit (automatic) attitudes toward physical activity. This dissociation suggests that, despite consciously reporting positive attitudes toward engaging in physical exercise, participants’ unconscious cognitive–emotional processes respond more negatively to physical activity-related stimuli. This mechanism has been interpreted as reflecting the impact of weight-related stigma, which plays a significant role in shaping attitudes toward physical activity among individuals with overweight and obesity. Furthermore, the observed correlation was associated with lower levels of self-esteem [17].

Although this represents a promising direction, further research is required to optimise and standardise movement-based posture intervention programmes that account for age, fitness level, and the individual needs of children and adolescents with overweight and obesity. Prospective studies indicate that body image plays a key mediating role in the relationship between obesity and subsequent depressive symptoms. In a cohort of children with obesity, lower body image evaluation in childhood was associated with higher levels of depressive symptoms during adolescence. The mediating effect of body image between obesity and the development of depressive symptoms was significant irrespective of sex, indicating that adolescents’ perceptions of their own bodies constitute an important determinant of depression risk. These findings suggest that interventions aimed at improving self-esteem and fostering a more positive body image may represent a crucial component in the prevention of depressive disorders among adolescents with excess body weight [24].

It is worth noting that the meta-analysis conducted by Moradi et al. did not identify a clear association between overweight or obesity and the risk of depressive and anxiety disorders in children and adolescents [10]. The authors included 25 cross-sectional studies and only three prospective studies, suggesting that the design and inclusion criteria of the analysed studies may have substantially influenced the observed outcomes. Consequently, findings regarding the association between obesity and depression or anxiety in the literature remain inconsistent, with discrepancies likely attributable to methodological differences, heterogeneity of study populations, variation in follow-up duration, and the use of diverse diagnostic instruments. The lack of prospective studies in the existing literature represents a significant limitation, which the present study aims to address. Employing a longitudinal design, it will enable a more robust understanding of the relationship between obesity and selected psychosocial and psychophysical outcomes.

The results of a one-year follow-up of children and adolescents with excess body weight will enable a multidimensional analysis of the consequences of obesity across biomechanical, psychological, and environmental domains. Longitudinal assessment of postural abnormalities will enable not only the determination of their prevalence but also the monitoring of progression and the identification of potential compensatory patterns within the musculoskeletal system. Concurrently, analysis of environmental and psychoemotional factors such as the children’s lifestyle, parental health awareness, level of physical activity, and emotional support will enable the evaluation of whether these variables constitute statistically significant predictors of postural disorders and impaired psychophysical functioning.

Integrating physical and psychosocial dimensions is essential for understanding the mechanisms underlying the accumulation of health burdens in children with obesity. The proposed study design addresses existing gaps in the literature and responds to the need for interdisciplinary care, involving collaboration among medical professionals, physiotherapists, dietitians, and psychologists. It also highlights the necessity of standardizing intervention programmes tailored to patients’ age, fitness level, and individual needs.

Interventions incorporating lifestyle modification, including physical activity, appropriate nutrition, and psychological support, have demonstrated beneficial effects on both physical health and psychological well-being in children, representing a comprehensive approach to the prevention and management of obesity-related consequences [25].

However, the present study is not without limitations. Due to the narrative nature of the review, a PRISMA flow diagram and a formal risk of bias assessment were not included. While systematic reviews employ predefined methodologies that reduce bias and enhance objectivity, narrative reviews are inherently more subjective. Systematic reviews also aim to include all relevant studies, thereby providing a more comprehensive synthesis of the available evidence. Consequently, the findings of a narrative review may be more susceptible to bias and should be interpreted with greater caution than those of systematic reviews, which represent a higher level of evidence in evidence-based medicine. Moreover, the studies included in this narrative review exhibit considerable heterogeneity in terms of sample size, sex distribution, and population characteristics and encompass both clinical and non-clinical groups. Some studies lack basic descriptive data on participants, including information on potential confounding factors such as socioeconomic status, physical activity levels, and developmental stage. In addition, there is a notable lack of longitudinal analyses, as the majority of included studies are observational and do not allow for causal inference. These limitations may significantly affect the generalisability of the findings. Therefore, further research is needed to better understand the relationships between body posture and psychophysical functioning in children with obesity.

## 5. Conclusions

In light of current evidence, overweight and obesity among children and adolescents remain a significant public health challenge, with increasing impacts on physical, psychological, and social functioning. Existing literature indicates that the consequences of excess body weight encompass both postural and movement impairments, as well as psychosocial burdens, including lower self-esteem and an elevated risk of depressive symptoms.

Effective preventive and therapeutic approaches require the integration of physical and psychosocial dimensions, taking into account the individual needs of the child, family environment, and level of physical activity. Interdisciplinary strategies combining health education, psychological support, appropriate physical activity, and physiotherapeutic interventions can not only mitigate the health consequences of obesity but also support the psychosocial development of children and adolescents.

At the same time, the analysis of existing research reveals substantial gaps, including a lack of longitudinal studies, methodological heterogeneity, and limited assessment of environmental and psychological factors. Future research should focus on long-term observations, standardization of intervention programs, and a deeper understanding of the mechanisms mediating the relationship between obesity and psychophysical functioning, thereby enabling more effective design of preventive and therapeutic strategies.

## Data Availability

No new data were created or analyzed in this study. Data sharing is not applicable to this article.

## Supplementary Materials

The following supporting information can be downloaded at: https://www.mdpi.com/article/10.3390/medicina62040779/s1, Supplementary Table S1. List of studies included in the review.
